# Key messages: global eye health

**Published:** 2018-02-08

## What progress has been made?

**Figure:**
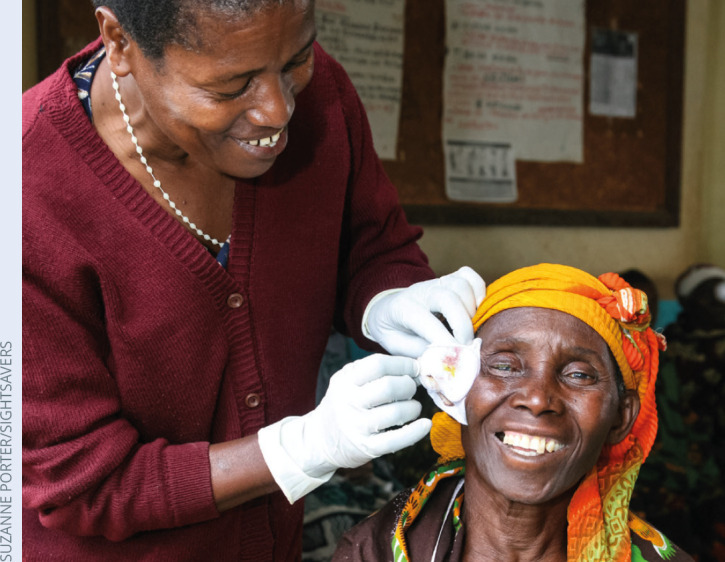

- The prevalence of blindness has decreased by 25%: from 4.6 per 1,000 people in 1990 to 3.4 per 1,000 people in 2015
- More people are receiving cataract surgery and implantation of an IOL is now routine, giving better post-operative vision
- Many people have received Mectizan for onchocerciasis, Zithromax for trachoma and Vitamin A supplementation for vitamin A deficiency, and blindness due to these infections and malnutrition has decreased

## What about human resources in eye health?

**Figure:**
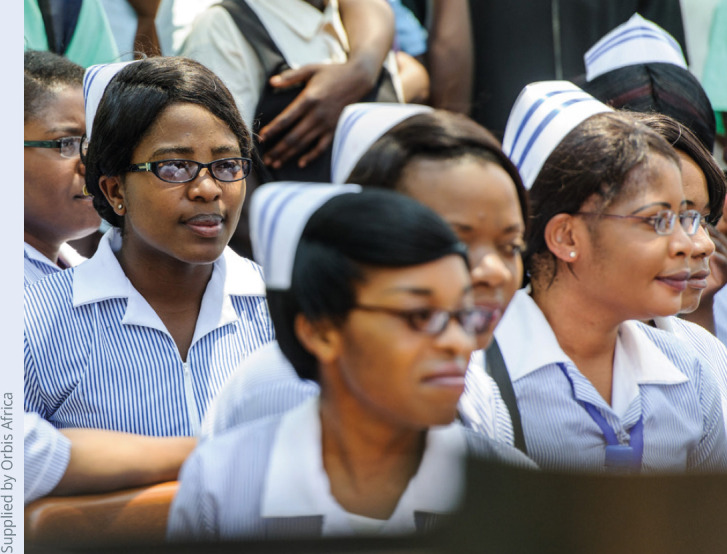

- There have been significant improvements worldwide in the training of ophthalmologists, optometrists and allied ophthalmic personnel (including ophthalmic nurses) around the world
- However, the number of eye health personnel in most African countries is well below the minimum recommended. There are insufficient training schools and graduates to meet the need for different types of eye health personnel

## What are the emerging disease challenges?

**Figure:**
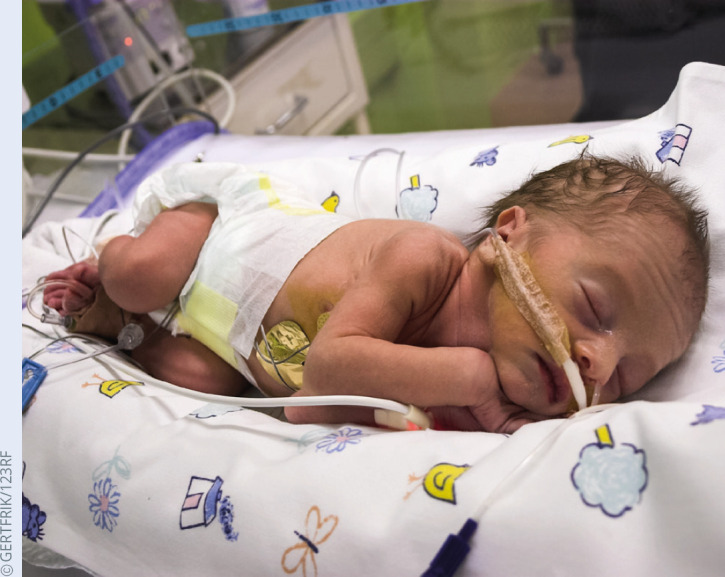

- Cataract is still the top cause of blindness
- Myopia is increasing among children and requires school screening programmes
- Glaucoma is the third cause of global blindness and cost-effective services are needed
- More people are developing diabetes and diabetic retinopathy, which requires treatment to prevent visual loss
- Retinopathy of prematurity is increasing among babies in middle-income countries

